# The Effects of Proprioceptive Neuromuscular Facilitation Pattern Kinesio Taping on Arm Swing, Balance, and Gait Parameters among Chronic Stroke Patients: A Randomized Controlled Trial

**DOI:** 10.3390/life14020242

**Published:** 2024-02-08

**Authors:** Seo-Jeong Moon, Sang-Yong Han, Dong-Hwan Park

**Affiliations:** Department of Physical Therapy, Graduate School, College of Health Science, Kyungnam University, Changwon-si 51767, Republic of Korea; tjwjd7834@gmail.com (S.-J.M.); hansangyong7744@gmail.com (S.-Y.H.)

**Keywords:** proprioceptive neuromuscular facilitation, kinesio tape, stroke, arm swing, balance, gait

## Abstract

(1) Background: This study aimed to investigate the effects of proprioceptive neuromuscular facilitation pattern kinesio taping on arm swing, balance, and gait parameters among chronic stroke patients. (2) Methods: Twenty-eight participants were randomized into proprioceptive neuromuscular facilitation pattern kinesio taping during gait training (*n* = 14) and gait training (*n* = 14) groups. The proprioceptive neuromuscular facilitation pattern kinesio taping during gait training group employed proprioceptive neuromuscular facilitation pattern kinesio taping during 15 min treadmill-based gait training five times a week for four weeks, while the gait training group underwent the same gait training without proprioceptive neuromuscular facilitation pattern kinesio taping. Arm swing angle was measured using the Image J program, static balance was assessed with an AMTI force plate, dynamic balance was evaluated through the Timed Up and Go test, and gait parameters were recorded using the GAITRite system and the Dynamic Gait Index. (3) Results: After 4 weeks of training, the proprioceptive neuromuscular facilitation pattern kinesio taping during gait training group exhibited significant improvements in all variables compared to the baseline (*p* < 0.05), whereas the gait training group did not show statistically significant differences in any variables (*p* > 0.05). (4) Conclusions: This study demonstrates the effectiveness of proprioceptive neuromuscular facilitation pattern kinesio taping during gait training in enhancing arm swing angle, balance, and gait parameters.

## 1. Introduction

Stroke affects approximately 115 million people worldwide, the majority of whom suffer from motor disabilities such as hemiplegia [1]. Two-thirds of stroke survivors still exhibit disabilities five years after the stroke’s onset [2]. One of these persistent disabilities is upper extremity motor impairment, with 50% of patients still experiencing upper-extremity functional problems even after 4 years [3,4]. Common symptoms of upper extremity disorders include muscle weakness or contractures, changes in muscle tone, and impaired motor control [5]. Balance disorders emerge in the acute stage in more than 80% of stroke patients, with over 40% experiencing persistent disruptions [6]. Stroke patients with balance disorders tend to bear less weight on their paralyzed leg, making it difficult to maintain balance in various environments [7]. These balance disturbances are associated with a decline in daily living activities and mobility and an increased risk of falls [8]. Gait disorders place the most significant limitation on daily activities for stroke patients [9]. After a stroke, most patients exhibit a slow gait speed, and abnormal gait patterns reduce endurance, thus limiting the distance they can walk [10]. These balance and gait impairments among stroke patients are associated with upper-extremity dysfunction and significantly limit the attainment of normal walking speed [11,12]. In a normal gait pattern, arm swing generates forward propulsion and aids in balance maintenance. However, stroke patients with impairments in upper-extremity function, balance, and gait may exhibit reduced or a complete lack of arm swing during walking, leading to difficulties in daily activities [13,14]. Therefore, the use of rhythmic movements of both the upper and lower limbs in rehabilitation therapy has been emphasized for the recovery of gait ability [15].

To enhance the function of the upper limbs and gait ability, rehabilitation interventions often implement Proprioceptive Neuromuscular Facilitation (PNF) techniques along with adjunctive methods such as the use of arm slings and kinesio taping [16,17,18]. Among these, PNF is particularly beneficial for improving function and range of motion among stroke patients, while the Bobath concept, which emphasizes the role of postural stability, is a widely adopted treatment method [19]. PNF is specifically used to treat both musculoskeletal and neurological patients, including those recovering from stroke, by stimulating the proprioceptors within the patient’s muscles, tendons, and joints [17]. Previous studies have demonstrated that the use of an arm sling during walking can increase the weight shift towards the affected side among stroke patients, leading to improved gait speed and an enhanced stance phase on the affected side [18]. Kinesio-taping therapy is another intervention method that facilitates or inhibits muscle function, provides joint stability, reduces pain, and rectifies the abnormal alignment of the trunk and scapula, thereby aiding the recovery of upper extremity function in stroke patients [16]. In a study involving stroke patients, the application of Kinesio tape to the upper extremity on the paralyzed side induced an improvement in gait parameters. A study on stroke patients reported that applying kinesio taping to a paralyzed upper extremity had a positive effect on gait by improving scapular movement and increasing arm swing [20].

Among these various treatment methods, the Proprioceptive Neuromuscular Facilitation Kinesio-Taping (PNF-KT) technique is used to induce the synergistic action of muscles by applying kinesio tape to muscle positions, similar to PNF patterns [21,22,23,24]. The pattern movements of PNF can be applied to various techniques such as rhythmic initiation, repeated contractions, rhythmic stabilization, and the hold–relax and contract–relax methods. According to the principles of PNF, the elongation position of the pattern is a target used to enhance muscle mobility and facilitate muscle activation [25]. Similarly, Kinesio taping is usually applied by elongating the tape [26]. Therefore, applying tape in the stretched position of the PNF pattern has been shown to be an extremely effective method for promoting muscle function [27]. In previous studies, it was found that applying PNF-KT to stroke patients resulted in improved movement and balance [24]. Similarly, the application of PNF-KT to stroke patients during treadmill training improved balance and gait by reducing muscle stiffness and enhancing muscle function [22].

Previous studies have demonstrated the potential of improving gait by applying PNF-KT to stroke patients; however, the corresponding evidence is limited and insufficient. Therefore, the aim of this study was to investigate the effects of applying PNF-KT to the upper extremities of stroke patients on arm swing, static and dynamic balance, and gait parameters. Additionally, we hypothesized that the group employing PNF-KT during gait training would exhibit differences in all indicators compared to the group solely performing gait training.

## 2. Materials and Methods

### 2.1. Participants

This study was conducted on chronic stroke patients admitted to H Hospital in Changwon City. A pilot test was performed to determine the minimum sample size. The results of the pilot test were used to conduct a G-power analysis in order to determine the sample size required to achieve a significance level of α = 0.05, a power of 0.8, and an effect size of 0.83. As a result of the analysis, it was determined that 12 subjects were needed for each group, and considering the dropout rate of 15%, 28 subjects were selected. This study recruited a total of 30 participants through recruitment advertisements posted on the hospital’s bulletin board. After applying exclusion criteria, 2 participants were excluded, resulting in a final sample size of 28 participants. The inclusion criteria for participants were as follows: (1) individuals with a confirmed neurologist-based diagnosis of stroke made at least 6 months post-diagnosis, (2) individuals who were able to walk 10 m without walking-assistive devices, and (3) individuals who scored 24 or higher on the Korean Mini-Mental State Examination (K-MMSE). The exclusion criteria were as follows: (1) individuals with fractures of the spine or upper extremities; (2) individuals who experienced severe shoulder pain; (3) individuals who had skin allergies to tape or experienced skin troubles due to tape; and (4) individuals with congenital joint deformities that could impact balance and gait. The purpose and methods of the experiment were fully explained to all patients. Participants fully understood the content of the experiment, participated voluntarily, and signed a written consent form. Additionally, all subjects were informed that they could withdraw from the experiment at any time for reasons such as pain, skin problems, or falls. Furthermore, the participants were informed that they could request to withdraw from the experiment or inquire about any other matters by contacting the research supervisor or research personnel at any time. This study was approved by the Kyungnam University Institutional Review Board (1040460-A-2023-027). This study was registered in Clinical Research Information Service (CRIS), and CRIS number is KCT0009090.

### 2.2. Study Design

**Figure 1:**
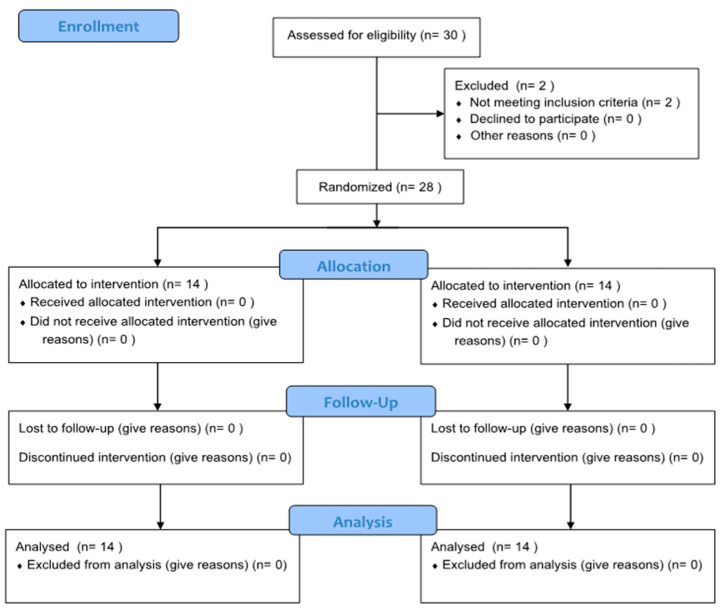
Flow chart of the experimental protocol.

This study was a randomized controlled trial. The experiment proceeded with the selection of the participants at random via Excel, with 14 individuals assigned to the PNF-KT group and an equal number assigned to the GT group. Additionally, the participants assigned to the PNF-KT group were assigned unique identification numbers ranging from P-01 to P-14, and the participants assigned to the GT group were assigned unique identification numbers ranging from G-01 to G-14. A flow chart of this study is shown in Figure 1. Interviews were conducted with a cohort of 28 participants to procure their general and medical attributes. Prior to the experiment, measurements were taken for arm swing angle, static and dynamic balance, and gait parameters. Throughout the examination phase, each participant underwent the same rehabilitative training following the everyday inpatient treatment plan. Each participant engaged in standard physiotherapy for a duration of 30 min per session, with the volume and variety of standard physiotherapy conforming to a protocol to maintain consistency among the participants in both groups. The first 10 min were assigned for active and passive range-of-motion exercises for the upper and lower extremities on the affected side. The following 10 min were set aside for weight-bearing exercises either in a standing or sitting posture. The final 10 min were dedicated for trunk movement and sit-to-stand exercises.

After standard physiotherapy, the PNF-KT group employed PNF-KT and performed treadmill-based gait training for 15 min each time, 5 times a week, a total of 20 times for 4 weeks. The GT group engaged in the same treadmill-based gait training but without employing PNF-KT. This study conformed to the CONSORT Guidelines.

### 2.3. Proprioceptive Neuromuscular Facilitation Pattern Kinesio Taping during Gait Training

**Figure 2:**
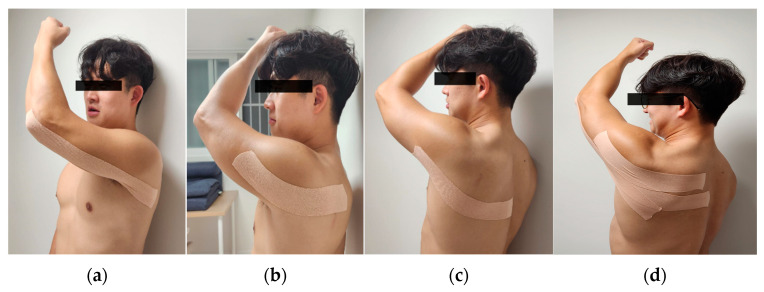
Application of Proprioceptive Neuromuscular Facilitation Pattern Kinesio tape to the upper extremities on the affected side: (**a**) proprioceptive neuromuscular Kinesio tape applied to the triceps brachii; (**b**) proprioceptive neuromuscular Kinesio tape applied to the posterior deltoid; (**c**) proprioceptive neuromuscular Kinesio tape applied to the latissimus dorsi; (**d**) appearance of all PNF-KT applications.

**Figure 3:**
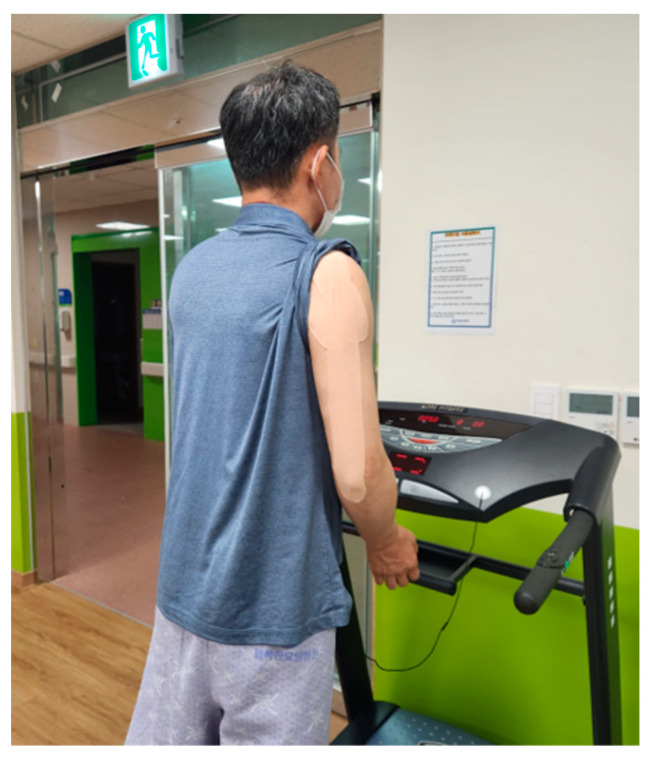
PNF-KT employed during treadmill-based gait training.

This study involved conducting gait training on stroke hemiplegia patients by applying Proprioceptive Neuromuscular Facilitation pattern Kinesio Tape to the upper extremity on the affected side (Figure 2). The upper extremity PNF patterns were named according to the position of the shoulder when a diagonal pattern was completed. The upper D1 flexion pattern (D1F) included the elevation, abduction, and upward rotation of the scapula and the flexion, adduction, and external rotation of the shoulder [28]. We used ATEX Kinesio^®^ Tape (ATEX medical, Co. Ltd., Gyeonggido, Republic of Korea) with a width of 5 cm. The method of attaching this tape was performed as follows, referring to previous studies [21,25]: (1) The subject assumed the D1 upper extremity pattern of the PNF pattern. (2) PNF-KT was employed to facilitate the use of the triceps brachii, posterior deltoid, and latissimus dorsi muscles on the affected side of an upper extremity. (3) The tape was stretched by 20% and attached along the length of the muscle distally to proximally to each muscle. Gait training was performed at a comfortable and safe speed using a treadmill under the supervision of one physical therapist (Figure 3).

### 2.4. Measurements

#### 2.4.1. Arm Swing Angle

The Image J program was used to measure the angle of arm swing while walking. Image J (version 18.0) is a Java-powered image-processing software product created by the National Institutes of Health. It is a cost-effective and portable system for motion analysis [29]. The method of measurement entails videotaping the subject as they walk on a treadmill for a duration of 5 min, then capturing the arm swing angles during the central 3 min period, excluding the initial and final minute, and subsequently transforming these recordings into images. Next, the images were analyzed using the Image J (version 18.0).

#### 2.4.2. Static Balance

For the assessment of static balance, evaluations were carried out for a span of 30 s in an eyes-open standing stance using an AMTI force plate (AMTI, Newton, MA, USA). The AMTI force plate was used by connecting a USB to a computer. The allowable load was 130 kg, and the size was 502 × 502 × 45 mm. The participants stood in a comfortable position on the force plate barefoot. The foot positions were recorded, and the feet were placed in the same position during re-examination The sway path of the COP (Center of Pressure) was measured for 30 s with the patients in a standing position with their eyes open while focusing on a point with a diameter of 15 cm located 3 m ahead. A data collection sampling frequency of 100 Hz and a measurement time of 30 s were used for each action [30]. The sway path (cm) of the COP was collected for each subject, and the total paths of movement in the mediolateral (ML) and anteroposterior (AP) directions were analyzed [31].

#### 2.4.3. Dynamic Balance

To assess dynamic balance ability, the Timed Up and Go test (TUG) was conducted. The TUG test involved several steps: (1) standing up from a sitting position in a chair, (2) walking for 3 m, (3) making a turn and coming back from a 3 m point, and (4) making a turn and sitting back down in a chair [32]. The results were measured three times, and the average value was used.

#### 2.4.4. Gait Speed

Gait speed was evaluated using the GAITRite system (GAITRite^®^ Electronic Walkway, CIR Systems Inc., Peekskill, NY, USA). The GAITRite system’s mat is 461 cm long and 88 cm wide. This piece of equipment had 13,824 sensors with a diameter of 1 cm arranged vertically at 1.27 cm intervals for collecting information on temporal and spatial variables. While a participant was walking comfortably on a gait analysis mat, the load exerted on their feet was recorded at a sampling rate of 80 Hz, and this information was sent to a computer via a serial interface cable [33]. In this study, the participants were positioned 2 m in front of the gait analysis mat and walked in a straight line at a comfortable speed while looking ahead, as directed via verbal cues given by the examiner. The measurements were conducted three times in total, and the average values were used. There was a one-minute rest period between each measurement [34]. 

#### 2.4.5. Dynamic Gait Index

This assessment tool was designed to determine the gait ability of stroke patients. It is designed to evaluate whether stroke patients can respond appropriately to external signals while walking and adjust their walking accordingly [35]. DGI consists of a total of 8 tasks: (1) walking 6 m, (2) changing walking speed, (3) turning one’s head left and right while walking, (4) moving one’s head up and down while walking, (5) turning 180 degrees and stopping while walking, (6) jumping over obstacles while walking, (7) crossing obstacles while walking, and 8) climbing and descending a flight of 4 stairs [36].

### 2.5. Statistical Analysis

Descriptive data analyses and statistical tests were performed using SPSS version 25.0 (SPSS, Inc., Chicago, IL, USA). Normality was checked using the Shapiro–Wilk test. Descriptive data were presented as mean ± standard deviation. Baseline demographic variables were compared between the groups by using an independent *t*-test for continuous data and the chi-square test of independence for categorical data. Differences between groups and time were analyzed using a mixed-model, two-by-two analysis of variance (ANOVA); the within-subjects variables were compared before and after the 4-week intervention, and the between-subjects variables were compared between the PNF-KT and the GT groups. Statistical significance was set at *p* < 0.05. Effect sizes were calculated to determine meaningful changes between groups, with an effect size of ≤0.20 indicating a small change; 0.50 indicating a moderate change; and 0.80 indicating a large change [37].

## 3. Results

Table 1 presents the general and medical characteristics and the results of normality testing for each group’s parameters. No significant differences were observed in the demographic data and parameters between the PNF-KT and GT groups.

**Figure 4:**
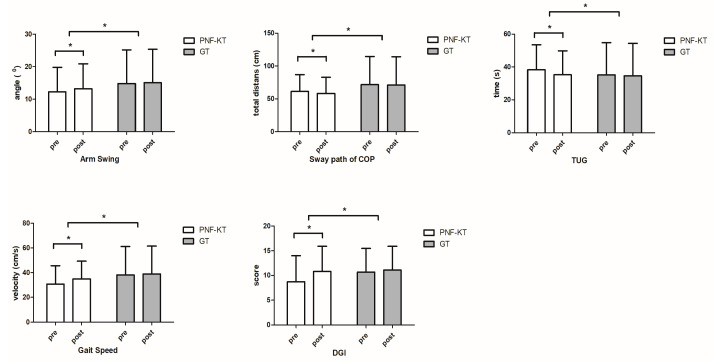
Comparison within and between groups according to pre- and post-intervention variables. PKT-GT, PNF-KT during gait training. GT, Only Gait Training. TUG, Timed Up and Go test. DGI, Dynamic Gait Index. * *p* < 0.05.

The measurement results are given in Table 2. Table 3 shows the differences in the values before and after the intervention for each group. And to improve comprehension of the statistical results, a comparison before and after intervention for each group is depicted in Figure 4. As a result of the ANOVA analysis, there were significant differences in the interactions, such as with respect to arm swing (*F* = 65.72, *ŋ*^2^ = 0.71, *p* < 0.05), the sway path of COP (*F* = 97.29, *ŋ*^2^ = 0.78, *p* < 0.05), TUG (*F* = 118.94, *ŋ*^2^ = 0.82, *p* < 0.05), gait speed (*F* = 136.3, *ŋ*^2^ = 0.84, *p* < 0.05), and DGI (*F* = 120.9, *ŋ*^2^ = 0.82, *p* < 0.05). The results of the independent t-test and the paired *t*-test are shown in Table 2 and Table 3. There was a significant difference in the arm swing angle, sway path of COP, TUG, gait speed, and DGI before and after the PNF-KT group intervention (*p* < 0.05). Regarding the pre–post difference, only the PNF-KT group showed a significant difference (*p* < 0.05), and a significant difference was also found in the comparison of the pre- and post-intervention difference values between the PNF-KT group and the GT group (*p* < 0.05).

## 4. Discussion

This study investigated the effects of PNF-KT on arm swing, balance, and gait parameters among patients with chronic stroke. The PNF-KT group employed PNF-KT and performed treadmill-based gait training for 15 min per session, 5 times a week, totaling 20 sessions over 4 weeks. The GT group underwent the same gait training but without incorporating PNF-KT.

There was a statistically significant difference in arm swing angle before and after intervention in the PNF-KT group, and there was also a significant difference compared to the GT group. Arm swing is a crucial component of human walking [38]. During walking, arm swing arises from the repetitive lengthening and shortening of the arm and shoulder muscles, particularly via the activation of the posterior deltoid and triceps brachii muscles when the arm swings back and forth [39]. Okayama et al. (2023) [40] observed an increase in arm swing during walking when KT was applied to the upper limbs. In PNF patterns, elongating the muscles is a method that enhances muscle extensibility, thereby facilitating easier muscle activation. KT is often applied in an elongated manner to achieve similar effects [25]. Therefore, applying tape in the stretched position of PNF patterns is suggested to be a highly effective method for promoting muscle function [27]. The PNF-KT method fosters functional muscle synergy, enhances functional movements, and leverages the elastic properties of KT to control the direction of muscle contraction and joint movement [23,41]. Previous studies substantiate the findings of this study. Thus, it is believed that the PNF-KT method activates the deltoid, triceps brachii, and latissimus dorsi muscles and governs the direction of muscle contraction and normal joint movement through the elasticity of KT, thereby increasing the arm swing angle while walking.

The sway path determined in the COP analysis revealed a significant difference in the PNF-KT group pre- and post-intervention compared to the GT group. In a previous study involving stroke patients, Arslan et al. (2021) [42] found a correlation between the function of the upper extremities and both postural control and balance. Declining upper extremity function in stroke patients hinders goal-reaching movements [43]. In order for the upper extremities to reach the target, the lever length of the arm must be increased by extending the shoulder and elbow, and the center of gravity of the arm must deviate from the center of gravity of the body [44]. This requires trunk stability to maintain balance and posture when moving the upper extremities [45]. However, in stroke patients, trunk stability is reduced, preventing the upper extremities from reaching the target and reducing situations wherein compensation for flexion, trunk rotation to compensate for insufficient distance, and shoulder and elbow length may vary [46]. The position and stability of the scapula influence the stability of the trunk. Given that the scapula links the upper extremity with the trunk, changing posture through scapular movement can enhance overall body function and balance [47,48,49,50]. Results from previous studies that had stroke patients perform isometric scapular exercises showed an improvement in trunk control and balance [51]. The scapula is involved in most upper-extremity movements, and the action of the muscles surrounding the scapula provides proximal stability and coordinates distal movement. Moreover, arm swing contributes to the symmetry in the alignment of the upper limbs and torso, enhancing the overall stability of the torso [52]. Therefore, as a result of this study, it is believed that the PNF-KT method improved the stability of the shoulder girdle and trunk by enhancing the movement of the affected upper extremity, thereby improving static balance.

In the TUG test analysis, the PNF-KT group showed a significant difference pre- and post-intervention compared to the GT group. Active arm movements play a crucial role in restoring balance by preventing body rotation when balance is compromised. Therefore, arm swing is essential for maintaining balance since it regulates movement when the body is imbalanced [53,54]. When balance is compromised, individuals typically use their upper limbs to adopt a safe posture. Stroke-related upper-limb impairment results in decreased arm mobility, posing challenges for balance maintenance [11,55]. A study involving stroke patients concluded that the motor function of a paralyzed arm post-stroke is correlated with postural balance. Arm movement is an imperative factor in restoring balance, and the motor function of a paralyzed arm post-stroke is associated with postural balance disorders [11,56]. Arm movement in balance restoration generates restorative torque that reduces the body’s angular momentum [57]. Arm movement generates an inertial moment in the body that opposes destabilizing external forces, thus reducing the angular velocity of falling [58]. In other words, arm movements affect balance and, specifically, contribute to the restoration of balance disrupted by body sway [59]. Several previous studies involving stroke patients have demonstrated that the motor function of the affected arm following stroke onset is correlated with postural balance [11,59,60]. Therefore, in this study, it is believed that the improved arm swing among stroke patients induced by the PNF-KT method positively impacted their balance ability.

The PNF-KT group showed statistically significant differences in the gait speed and DGI analysis before and after intervention compared to the GT group. In a study involving stroke patients, applying the PNF D1 pattern to the paralyzed upper limb increased muscle activity in key gait muscles, including the vastus medialis, tibialis anterior, rectus femoris, and gastrocnemius [61,62]. During gait, restricted arm movements affect the gait patterns of both healthy adults and stroke patients. There is indirect evidence supporting a correlation between upper- and lower-limb muscle activation patterns [60]. Upper extremity movement is crucial in human gait and balance control, and recent research emphasizes addressing arm movements during gait rehabilitation for stroke patients, as arm swing influences the muscles of the lower extremities [59,60,63,64]. In a previous study, Yavuzer and Ergin (2002) [18] analyzed walking patterns by applying arm slings to stroke patients to aid their ability to walk resulting in improvements in walking speed and stance phase on the paraplegic side. Hwang et al. (2015) [65] applied an elastic arm sling to stroke patients to reduce spasticity, which resulted in significant improvements in gait speed, stride length, and bilateral step length. A study specifically investigating stroke patients reported an improvement in gait parameters with the application of Kinesio tape to the upper extremity of the paralyzed side. These results suggest that rhythmic arm movements affect body stabilization by coordinating the regulation of body angular momentum [20]. Additionally, a recent study reported that voluntary arm shaking while walking induces activity in the corticospinal tract and reflex responses, thereby inducing activity in lower limb muscles [66]. In this study, proprioceptive neuromuscular Kinesio tape was applied to the upper extremities of stroke patients to analyze gait, and results similar to those presented in previous studies were observed. Therefore, it is believed that PNF-KT improves trunk stability by controlling the angular movements that occur during walking and contributes to increased lower limb muscle activity and the creation of a stable gait pattern, thereby improving gait speed and ability.

This study has several limitations. First, the study period was short, which made it difficult to assess the long-term effects of PNF-KT. Therefore, further research is needed to examine the sustainability of PNF-KT. Second, no follow-up was conducted, which pre-vented us from investigating the carryover effect of PNF-KT. Finally, the age range of the participants, from 40 to 80 years old, limits the generalizability of the findings to a broader age range. Further studies are required to address these limitations and to investigate the sustainability of the effects of PNF-KT.

## 5. Conclusions

This study investigated the effects of proprioceptive neuromuscular Kinesio tape applied to the upper extremities on arm swing, static and dynamic balance, and gait parameters among patients with chronic stroke. The results showed that the PNF-KT group had significant improvements in arm swing, static and dynamic balance, and gait parameters compared to the GT group. These findings suggest that PNF-KT is a treatment method that can enhance arm swing during walking and improve balance and walking ability in the clinical rehabilitation of stroke patients.

The results of this study on PNF-KT provide foundational data for research on taping, which is considered a therapeutic method that can contribute to the functional recovery of stroke patients.

## Figures and Tables

**Table 1:** General and medical characteristics and the results of the parameter normality test (*n* = 28).

Category	PNF-KT (*n* = 14)	GT (*n* = 14)	*p*
Sex (male/female)	7/7	8/6	0.45 ^a^
Age (years)	60.3 ± 7.5	59.2 ± 8.2 ^a^	0.988 ^b^
Height (cm)	164.1 ± 7.3	162.3 ± 8.9	0.987 ^b^
Weight (kg)	61.8 ± 11.1	61.6 ± 15.1	1.000 ^b^
Affected side (Right/Left)	8/6	8/6	0.45 ^a^
Stroke type (Infarction/Hemorrhage)	5/9	8/6	0.705 ^a^
Onset (month)	35.3 ± 16.1	28.5 ± 13.5	1.000 ^b^
K-MMSE (score)	26.7 ± 2.2	26.4 ± 1.9	0.712 ^b^
Arm Swing (°)	12.3 ± 7.5	14.8 ± 10.3	0.466 ^b^
Sway path of COP (cm)	61.1 ± 25.6	71.7 ± 42.8	0.436 ^b^
TUG (sec)	38.3 ± 15.3	35.2 ± 19.6	0.649 ^b^
Gait speed (cm/s)	30.6 ± 15.0	38.2 ± 23.0	0.316 ^b^
DGI (score)	8.7 ± 5.3	10.6 ± 4.8	0.323 ^b^

Data are expressed as mean ± SD or *n.* PNF-KT, PNF-KT during gait training. GT, Only Gait Training. K-MMSE, Korean Mini-Mental State Examination. TUG, Timed Up and Go test. DGI, Dynamic Gait Index. ^a^ Obtained using the χ^2^ test. ^b^ Obtained using an independent *t*-test.

**Table 2:** Comparison of parameters pre- and post-intervention for each group (*n* = 28).

Parameters	PNF-KT (*n* = 14)			GT (*n* = 14)		
	Pre	Post	*p*	Pre	Post	*p*
Arm Swing (°)	12.3 ± 7.5	13.2 ± 7.6	0.001 *	14.8 ± 10.3	15.1 ± 10.3	0.075
Sway path of COP (cm)	61.1 ± 25.6	58.2 ± 24.7	0.001 *	71.7 ± 42.8	71.1 ± 43.0	0.075
TUG (sec)	38.3 ± 15.3	35.3 ± 14.5	0.009 *	35.2 ± 19.6	34.6 ± 19.7	0.142
Gait speed (cm/s)	30.6 ± 15.0	34.9 ± 14.4	0.001 *	38.2 ± 23.0	38.9 ± 22.5	0.087
DGI (score)	8.7 ± 5.3	10.8 ± 5.1	0.003 *	10.6 ± 4.8	11.1 ± 4.8	0.793

Data are expressed as mean ± SD. PKT-GT, PNF-KT during gait training. GT, Only Gait Training. TUG, Timed Up and Go test. DGI, Dynamic Gait Index. * *p* < 0.05.

**Table 3:** Comparison of differences pre- and post-intervention between groups (*n* = 28).

Parameters	PNF-KT (*n* = 14)	GT (*n* = 14)	*p*	ES
	Difference (Post–Pre)	Difference (Post–Pre)		
Arm Swing (°)	0.9 ± 0.6	0.3 ± 0.6	0.004 *	1.17
Sway path of COP (cm)	−2.9 ± 1.6	−0.6 ± 1.2	0.008 *	1.65
TUG (sec)	−3.0 ± 3.6	−0.6 ± 1.3	0.026 *	0.97
Gait speed (cm/s)	4.2 ± 3.6	0.8 ± 1.6	0.005 *	1.3
DGI (score)	2.1 ± 2.1	0.4 ± 1.1	0.019 *	1.03

Data are expressed as mean ± SD. Difference, posttest-pretest. PNF-KT, PNF-KT during gait training. GT, Only Gait Training. TUG, Timed Up and Go test. DGI, Dynamic Gait Index. ES, effect size. * *p* < 0.05.

## Data Availability

The data used in this study are available upon request from the corresponding author. The data are not publicly available due to privacy or ethical considerations.
